# Sentinel Lymph Node Positivity and Utility of Frozen Section Diagnosis following Neoadjuvant Therapy in Patients with Clinically Node-Negative, Hormone Receptor-Positive, HER2-Negative Breast Cancer

**DOI:** 10.1245/s10434-026-19783-y

**Published:** 2026-05-14

**Authors:** Kerollos Nashat Wanis, Rrita Krasniqi, Susie X. Sun, Erika Resetkova, Rosa F. Hwang, Kelly K. Hunt, Rosalind P. Candelaria, Lei Huo, Puneet Singh

**Affiliations:** 1 Department of Breast Surgical Oncology, The University of Texas MD Anderson Cancer Center, Houston, TX USA; 2 Department of Biostatistics, The University of Texas MD Anderson Cancer Center, Houston, TX USA; 3 Department of Health Services Research, The University of Texas MD Anderson Cancer Center, Houston, TX USA; 4 Institute for Data Science in Oncology, The University of Texas MD Anderson Cancer Center, Houston, TX USA; 5 Department of Pathology, The University of Texas MD Anderson Cancer Center, 1515 Holcombe Blvd., Houston, 77030 TX USA; 6 Department of Breast Imaging, The University of Texas MD Anderson Cancer Center, Houston, TX USA

## Abstract

**Background:**

Frozen section evaluation can be used for intraoperative diagnosis of sentinel lymph node (SLN) metastases. When completion axillary lymph node dissection is planned owing to positive SLNs following neoadjuvant chemotherapy (NACT), frozen section may be considered to facilitate completion of axillary surgery at the index operation. The utility of intraoperative SLN assessment with frozen section in patients with hormone receptor-positive (HR^+^) breast cancer depends on the prevalence of SLN positivity and frozen section accuracy.

**Patients and Methods:**

We conducted a single-institution observational cohort study including patients with noninflammatory, cN0, HR^+^, defined as either estrogen receptor-positive (ER^+^) and/or progesterone receptor-positive (PR^+^), HER2-negative breast cancer treated with NACT between 2015 and 2021. We estimated the prevalence of SLN positivity and the diagnostic test characteristics of SLN frozen section.

**Results:**

Of 145 eligible patients, 47 (32.4%; 95% confidence interval [CI]: 24.9, 40.7) had SLN metastases. Positive SLNs were more frequent among those with ER^+^ PR^+^ tumors compared with ER^+^ PR^−^ or ER^−^ PR^+^ tumors, larger clinical T category, and invasive lobular histology. Among 115 patients with SLNs assessed by frozen section, the true prevalence of metastases was 31.3% (95% CI: 23.0, 40.6), while frozen section identified only 17.4% (95% CI: 11.0, 25.6) as positive. Frozen section sensitivity was 55.6% (95% CI: 38.1, 72.1), and specificity was 100% (95% CI: 95.4, 100.0). Sensitivity for micrometastases or isolated tumor cells was limited (23.5%; 95% CI: 6.8, 50.0).

**Conclusions:**

In patients with cN0 HR^+^, HER2-negative breast cancer, SLN positivity after NACT varies and is more common in high-risk subgroups of this population. Frozen section has modest sensitivity overall and is more limited for the diagnosis of low-volume nodal disease. Use of frozen section may be warranted where avoiding reoperation is a priority.

Intraoperative frozen section can provide useful diagnostic information with implications for real-time surgical decision-making. In patients with breast cancer undergoing sentinel lymph node (SLN) biopsy, intraoperative frozen section assessment may reveal the presence of nodal metastases.^1^ This information can be instructive in the setting of prior neoadjuvant systemic therapy, where the current standard practice recommendation includes completion axillary lymph node dissection (ALND) when any lymph node metastases are diagnosed.^2^

When frozen section assessment accurately diagnoses lymph node metastases in the setting of prior neoadjuvant therapy, surgeons can use this information to complete all recommended axillary surgery during a single operation, potentially reducing total anesthetic time and minimizing delays to adjuvant therapies. However, the success of this approach depends on the accuracy of frozen section diagnosis. The reported sensitivity of frozen section diagnosis for SLN metastases varies widely in different studies, ranging from 44 to 100% in a previous review.^3^ Small-volume metastasis, i.e., micrometastasis and isolated tumor cells, and lobular tumor morphology, are associated with lower accuracy of intraoperative SLN evaluation.^3–5^ Some studies have also reported that false-negative rates for intraoperative SLN diagnosis are higher in patients who have undergone treatment with neoadjuvant chemotherapy.^6,7^ Common factors negatively affecting the identification of residual metastasis include fibrosis, deposit of histiocytes, reactive changes in endothelial cells, and alteration of tumor morphology in SLNs due to therapy.

If intraoperative SLN assessment has limited specificity, the results can lead to unnecessary use of completion ALND in patients without SLN positivity, causing potential harm to the patient. Conversely, when sensitivity is poor, patients and surgeons may be falsely reassured and the abovementioned advantages will not be realized. In a prior study including patients with triple-negative or HER2-positive breast cancer following neoadjuvant systemic therapy, we estimated that sentinel lymph node frozen section has excellent specificity but modest sensitivity.^8^

Even when frozen section examination of SLNs is accurate, its routine use can be costly, time-consuming, require substantial pathologist experience, and may consume portions of the specimen, impacting the accuracy of permanent histological evaluation.^9–11^ Thus, the decision to perform frozen section evaluation should depend, in part, on the pre-evaluation probability of SLN positivity. While this probability is low in patients with triple-negative and HER2-positive breast cancer,^8^ it may be substantially higher in patients with hormone receptor-positive (HR^+^), HER2-negative breast cancer who are more likely to have advanced disease if undergoing neoadjuvant systemic therapy, and less likely to experience a pathologic complete response to these therapies.^12,13^

To inform decision-making, in this study, we estimate the prevalence of SLN positivity in patients with cN0, HR^+^, HER2-negative breast cancer treated with neoadjuvant systemic therapy and the test characteristics of frozen section examination in this setting.

## Patients and Methods

### Study Population and Variables

Using a prospectively maintained institutional database, we identified and collected data on a cohort of patients diagnosed with cN0, HR^+^, HER2-negative breast cancer between January 2015 and November 2021 who were treated with neoadjuvant chemotherapy followed by surgery, including sentinel lymph node biopsy. HR^+^ was defined as either estrogen receptor (ER)- and/or progesterone receptor (PR)-positive. The majority of the included patients had their pretreatment work-up and definitive surgery at MD Anderson Cancer Center where dedicated axillary ultrasound was routinely performed during initial clinical staging. A minority of patients were included even if their diagnostic assessment and surgery were performed outside of our institution, and, for these patients, their staging work-up was performed according to the local standards at their treating institutions. We excluded patients who had inflammatory breast cancer, did not undergo SLN biopsy, those who received neoadjuvant endocrine therapy or targeted therapies (e.g., poly (ADP-ribose) polymerase inhibition) without chemotherapy, did not undergo surgery following systemic therapy, or who did not have complete pathologic information available. The University of Texas MD Anderson Cancer Center Institutional Review Board approved this cohort study.

We collected data on the clinical and pathologic characteristics of the patients and their tumors. This included data on age at diagnosis, sex, race and ethnicity, clinical T category, tumor histology, and tumor subtype based on immunohistochemical analysis of receptor status. To categorize ER and PR status, ≥ 10% staining of the tumor cells by immunohistochemistry (IHC) was considered positive for each receptor. Tumors were considered HER2-negative if they were 0 or 1+ by IHC or 2+ by IHC and nonamplified by an in situ hybridization method.

Surgical pathology variables included breast primary histology, SLN status, and axillary lymph node status if completion ALND was performed. When intraoperative frozen section was performed, the intraoperative diagnosis was recorded. SLN pathological variables included the number of nodes and size of SLN metastases. For patients who had SLN biopsy performed at our institution, keratin immunohistochemistry was routinely used on permanent sections to assist with the identification of small metastasis. If ALND was performed, the number of involved axillary lymph nodes was recorded. Patients were considered to have had a pathologic complete response (pCR) when their final pathology was ypT0N0 or ypTisN0.

For the subgroup of patients who underwent work-up and definitive breast surgery at MD Anderson Cancer Center, we also collected information on post-neoadjuvant, preoperative radiological assessment of primary tumor response, which was defined as progression if the radiologist reported an increase in size of the tumor, partial response if the radiologist reported a decrease in size, or stability if the tumor was reported as unchanged.

### Statistical Analysis

We estimated the prevalence of SLN positivity for the entire cohort of patients with cN0 HR^+^, HER2-negative breast cancer who received neoadjuvant systemic therapy and underwent breast surgery with SLN biopsy. We further computed the prevalence of SLN positivity in subgroups defined by tumor characteristics and response to therapy on imaging, using Fisher’s exact test *p*-values to quantify the strength of the evidence for associations.

We estimated the diagnostic test characteristics of SLN frozen section examination in the subset of patients in which frozen section assessment was performed. In this subset, we computed the apparent and true prevalence of SLN positivity. The apparent (test) prevalence is the probability of frozen section positivity, regardless of the final pathology result, and the true prevalence is the probability of positive SLNs on final pathology. We separately computed the test characteristics for diagnosis of isolated tumor cells and micrometastases versus macrometastases. Exact binomial confidence intervals were computed for these estimates.

## Results

### Pathologic Response and SLN Positivity Following Neoadjuvant Therapy

There were 145 patients who met eligibility criteria. The mean age at diagnosis was 47.9 years (standard deviation: 12.1 years). Most patients had clinical T category 2 or 3 tumors (83.5%) and invasive ductal carcinoma (IDC) histology (84.1%). The most common receptor combination was ER^+^ PR^+^ (48.3%), followed by ER^+^ PR^−^ (44.1%). The patient and tumor characteristics for the overall cohort are presented in Table 1.

**Table 1:** Cohort characteristics

Variable	Level	Overall	Frozen sections performed
No.		145	115
Age at diagnosis, mean (SD), years		47.9 (12.1)	47.4 (12.3)
Sex	Female	145 (100.0)	115 (100.0)
Race and ethnicity	Black	20 (13.8)	17 (14.8)
	Hispanic	19 (13.1)	17 (14.8)
	Asian/Pacific Islander	14 (9.7)	11 (9.6)
	White	88 (60.7)	68 (59.1)
	Other	4 (2.8)	2 (1.7)
Clinical T category	T1	17 (11.7)	12 (10.4)
	T2	81 (55.9)	64 (55.7)
	T3	40 (27.6)	32 (27.8)
	T4a–c	7 (4.8)	7 (6.1)
Histology	IDC	122 (84.1)	100 (87.0)
	ILC	17 (11.7)	12 (10.4)
	Mixed IDC/ILC	2 (1.4)	1 (0.9)
	Other	4 (2.8)	2 (1.7)
Receptor status	ER^+^ PR^+^, HER2-negative	70 (48.3)	53 (46.1)
	ER^+^ PR^−^, HER2-negative	64 (44.1)	52 (45.2)
	ER^−^ PR^+^, HER2-negative	11 (7.6)	10 (8.7)

Numbers are No. (%)

*IDC* invasive ductal carcinoma, *ILC* invasive lobular carcinoma, *ER* estrogen receptor, *PR* progesterone receptor, *HER2* human epidermal growth factor receptor 2, *SD* standard deviation

Final surgical pathology demonstrated pCR in 25 (17.2%, 95% confidence interval [CI]: 11.5, 24.4) patients. pCR was more common in patients with ER^+^ PR^−^ or ER^−^ PR^+^ tumors than those with ER^+^ PR^+^ tumors (21 of 75 [28.0%] versus 4 of 70 [5.7%]; Fisher's exact test *p*-value < 0.001).

Of the 145 patients, 47 had positive SLNs (32.4%; 95% CI: 24.9, 40.7) on final surgical pathology. SLN positivity was more common in patients with more advanced clinical T category, those with ER^+^ PR^+^ tumors compared with those with ER^+^ PR^−^ or ER^−^ PR^+^ tumors, and those with invasive lobular carcinoma (ILC) histology. The probability of SLN positivity stratified by these factors is presented in Table 2. Patients with a combination of these high-risk factors were more likely to have positive SLNs: 20 of 26 (76.9%; 95% CI: 56.4, 91.0) patients with ER^+^ PR^+^ and cT3 or cT4 tumors had positive SLNs. In contrast, only 2 of 41 (4.9%; 95% CI: 0.6, 16.5) patients with ER^+^ PR^−^ or ER^−^ PR^+^ and cT2 tumors had positive SLNs (Table 3 presents the probability of SLNB stratified by cT category and receptor status). Irrespective of histology, patients with ER^+^ PR^+^ tumors were likely to have positive SLNs: 9 of 14 (64.3%; 95% CI: 35.1, 87.2) of those with ILC and 31 of 56 (55.4%; 95% CI: 41.5, 68.7) of those with IDC or other histologies had positive SLNs (Table 3).

**Table 2:** Sentinel lymph node biopsy final pathology result by approximated receptor status, clinical T category, and histology

Variable	Level	Pos	Neg
No.		47	98
Receptor status^a^	ER^+^ PR^−^ or ER^−^ PR^+^	7 (9.3)	68 (90.7)
	ER^+^ PR^+^	40 (57.1)	30 (42.9)
Clinical T category^b^	T1	4 (23.5)	13 (76.5)
	T2	19 (23.5)	62 (76.5)
	T3 or T4a–c	24 (51.1)	23 (48.9)
Histology^c^	ILC	10 (58.8)	7 (41.2)
	IDC or other	37 (28.9)	91 (71.1)

^a^Fisher’s exact test *p*-value < 0.001

^b^Fisher’s exact test *p*-value = 0.005

^c^Fisher’s exact test *p*-value = 0.024

Numbers are No. (%)

*IDC* invasive ductal carcinoma, *ILC* invasive lobular carcinoma, *ER* estrogen receptor, *PR* progesterone receptor, *Pos* positive, *Neg* negative

**Table 3:** Sentinel lymph node biopsy final pathology result in low and high clinical T categories, and in ILC versus non-ILC histology categories, by receptor status

	cT1 or cT2		cT3 or cT4a–c		ILC		IDC or other	
Receptor status^a,b^	SLNB positive	SLNB negative	SLNB positive	SLNB negative	SLNB positive	SLNB negative	SLNB positive	SLNB negative
ER^+^ PR^+^	20 (45.5)	24 (54.5)	20 (76.9)	6 (23.1)	9 (64.3)	5 (35.7)	31 (55.4)	25 (44.6)
ER^+^ PR^−^ or ER^−^ PR^+^	3 (5.6)	51 (94.4)	4 (19.0)	17 (81.0)	1 (33.3)	2 (66.7)	6 (8.3)	66 (91.7)

^a^Fisher’s exact test *p*-value < 0.001 in patients with cT1 or cT2 tumors and < 0.001 in patients with cT3 or cT4a–c tumors

^b^Fisher’s exact test *p*-value = 0.54 in patients with ILC tumors and < 0.001 in patients with IDC or other tumors

Numbers are No. (%)

*SLNB* sentinel lymph node biopsy, *ILC* invasive lobular carcinoma, *IDC* invasive ductal carcinoma, *ER* estrogen receptor, *PR* progesterone receptor

There were 110 patients who underwent preoperative imaging at our institution to assess response to systemic therapy. The majority (101 patients [91.8%]) had evidence of tumor response on preoperative imaging after completion of neoadjuvant therapy. The probability of SLN positivity stratified by preoperative disease status is presented in Table 4.

**Table 4:** Sentinel lymph node biopsy final pathology result among those with post-neoadjuvant, preoperative imaging

Variable	Level	Positive	Negative
No.		34	76
Disease status^a^	Progression	0 (0.0)	5 (100.0)
	Response	33 (32.7)	68 (67.3)
	Stable	1 (25.0)	3 (75.0)

^a^Fisher’s exact test *p*-value = 0.38

Numbers are No. (%)

Post-neoadjuvant, preoperative imaging was considered to show progression if the radiologist reported an increase in the size of the tumor, response if the radiologist reported a decrease in size, or stability if the tumor was reported as unchanged

Among the 47 patients with positive SLNs, 35 patients (74.5%) had one positive SLN, 9 patients (19.1%) had two positive SLNs, and 3 patients (6.4%) had three positive SLNs. Overall, 32 patients (68%) underwent completion ALND with a median of 15 additional lymph nodes removed (interquartile range: 11, 21) with 8 of the 32 (25.0%) having additional positive lymph nodes. Those eight patients had a median of 2 (interquartile range: 1, 6) additional positive lymph nodes.

### Frozen Section Test Characteristics

Intraoperative frozen section assessment was performed in 115 of 145 (79.3%) patients. The patient and tumor characteristics for this cohort are presented in Table 1. The number of patients with positive SLNs in this subset was 36 (true prevalence: 31.3%; 95% CI: 23.0, 40.6), while frozen section examination was positive in 20 patients (apparent prevalence: 17.4%; 95% CI: 11.0, 25.6). The sensitivity of frozen section diagnosis was 55.6% (95% CI: 38.1, 72.1), the specificity was 100% (95% CI: 95.4, 100.0), the positive predictive value was 100% (95% CI: 83.2, 100.0), and the negative predictive value was 83.2% (95% CI: 74.1, 90.1). The sensitivity for isolated tumor cells or micrometastases was 23.5% (95% CI: 6.8, 50.0), and for macrometastases it was 84.2% (95% CI: 60.4, 96.6). There was not strong evidence for variability in sensitivity when comparing subsets of the cohort on the basis of receptor status, clinical T category, or histology. Of the 16 patients with a false-negative frozen section examination, 3 patients (18.8%) had macrometastases, 9 (56.3%) had micrometastases, and 4 (25.0%) had isolated tumor cells on final pathology.

### MD Anderson Cancer Center Subcohort

In sensitivity analysis, we considered the cohort of 120 patients who underwent diagnostic work-up and definitive surgery at MD Anderson. The probability of SLN positivity was similar in this subcohort compared with the entire study cohort (38 of 120 patients [31.7%; 95% CI: 23.5, 40.8]). The probability of SLN positivity stratified by tumor characteristics and frozen section test characteristics was also similar for this subcohort. In this subcohort, 52 (43.3%) patients had a suspicious finding on axillary imaging and 51 (42.5%) of them had an axillary lymph node biopsy prior to treatment, with benign findings. The probability of SLN positivity was 27.5% (14 of 51) in those who had a pretreatment axillary lymph node biopsied, and 34.8% (24 of 69) in those who did not (Fisher’s exact test *p*-value: 0.43).

## Discussion

We estimated that the prevalence of SLN positivity is high, at 32.4%, in the overall population of patients with HR^+^, HER2-negative tumors who are cN0 at diagnosis and are treated with neoadjuvant chemotherapy. Patients with ER^+^ PR^+^ tumors, those with more advanced clinical T category tumors, and those with invasive lobular histology had an even higher risk of SLN positivity following neoadjuvant chemotherapy. In this population, the estimated sensitivity of intraoperative frozen section diagnosis was modest at 55.6%, while specificity was 100%. The high prevalence of SLN positivity was also observed in a sensitivity analysis conducted using a subcohort of patients whose diagnostic work-up included dedicated pretreatment axillary ultrasound.

Although we estimated a high prevalence of SLN positivity in the overall population of patients with HR^+^ HER2^−^ tumors, some subsets of patients were less likely to have SLN metastases. In particular, patients with ER^+^ PR^−^ or ER^−^ PR^+^ and less advanced clinical T category tumors had a low prevalence of SLN positivity, similar to that observed in patients with triple-negative or HER2-positive breast cancer following neoadjuvant systemic therapy.^8^ This finding is consistent with the parallel finding of a higher probability of pCR in patients with ER^+^ PR^−^ or ER^−^ PR^+^ tumors. Prior studies support the high prevalence of SLN positivity and low likelihood of pCR in patients with HR^+^, HER2-negative tumors.^12,14–17^ Further, other studies have reported that lower PR percent positivity is associated with a higher likelihood of pCR in patients with HR^+^, HER2-negative tumors and cN0 or cN1 nodal category, consistent with luminal B biology.^18–20^ Our study supports and extends prior work by finding clinically meaningful heterogeneity in SLN positivity across categories defined by clinical and pathological characteristics for patients with cN0 HR^+^ tumors treated with neoadjuvant chemotherapy.

For patients who are likely to have SLN positivity following neoadjuvant systemic therapy, frozen section may be a useful tool to establish the diagnosis of SLN metastasis intraoperatively, facilitating the completion of ALND at the index operation. We estimated that frozen section has modest sensitivity (55.6%) for any SLN metastases and 100% specificity. For patients with ER^+^ PR^+^, HER2-negative breast cancer undergoing neoadjuvant systemic therapy, who we estimated had a 57.1% prevalence of SLN positivity, the estimated probability of a positive frozen section result, thus facilitating all necessary axillary surgery during a single operation, is 31.7%. Therefore, if all patients with positive SLNs undergo completion ALND, approximately three patients with ER^+^ PR^+^, HER2-negative breast cancer would need to undergo frozen section assessment to avoid one reoperation for completion ALND. In many clinical settings, the balance of patient values and costs may favor frozen section examination of SLNs for these patients with the goal of avoiding reoperation. Conversely, risks of ALND, such as lymphedema, may warrant performing completion ALND in the setting of a positive SLN at a second operation to facilitate immediate lymphatic reconstruction.^21,22^

Despite a modest overall sensitivity, we estimated that frozen section diagnosis has limited sensitivity for small-volume metastases (23.5%). However, the prognostic implication of minimal residual axillary disease following neoadjuvant systemic therapy is debatable, with conflicting evidence.^23–26^ In many cases with small-volume metastases, multidisciplinary teams may decide against completion ALND on the basis of the balance of individual patient factors. Indeed, even in patients with more extensive nodal disease, omission of completion ALND following neoadjuvant therapy has become more common in recent years.^27–30^ Ongoing studies, such as the Alliance A011202 (NCT01901094) trial, are expected to soon report results clarifying the oncologic implications of ALND omission in patients with residual nodal disease.^31,32^ Although the inclusion criteria for the Alliance A011202 trial are different than our study cohort, it is possible that the results may be extrapolated to patients with cN0 disease who are found to have nodal disease after neoadjuvant chemotherapy.

Our study reports findings that complement a recently published study estimating the prevalence of SLN positivity and frozen section test characteristics in patients with triple-negative or HER2-positive breast cancer.^8^ In contrast to those populations, we found that, in the aggregate, patients with HR^+^ breast cancer are much more likely to have positive SLNs, with some small subsets of patients (e.g., patients with small ER^+^ PR^−^ or ER^−^ PR^+^ tumors) having a lower prevalence. The SLN positivity prevalences reported in this study reflect estimates of the expected prevalence in a population of patients selected for neoadjuvant therapy and seen at our institution. Thus, the overall cohort size is limited as patients with cN0, HR^+^, HER2-negative breast cancer are often not recommended neoadjuvant chemotherapy. Importantly, this population may be different from that selected for upfront surgery versus neoadjuvant therapy at other centers,^33,34^ which may impact the interpretation of these results at institutions where neoadjuvant therapy is more or less likely to be recommended for patients with HR^+^ breast cancer and may be based on genomic assays. To help guide decision-making, we reported associations between the values of various preoperative variables and the prevalence of SLN positivity. While SLN positivity was strongly associated with certain variables (e.g., receptor status) in our study, the number of patients in some subgroups was small, and these estimates should be interpreted with the appropriate degree of uncertainty.

In conclusion, we estimated that SLN positivity was common in patients with cN0, HR^+^, HER2-negative breast cancer following neoadjuvant chemotherapy, with ER^+^ PR^+^ tumors, more advanced clinical T category, and invasive lobular histology being associated with a higher prevalence of SLN positivity. These findings may be particularly informative in counseling patients preoperatively on prevalence of SLN positivity. In settings where reoperation for completion ALND for positive SLNs is undesirable, use of frozen section may be warranted to complete all axillary surgery in one operation.
